# Exploring the Role of C-C Motif Chemokine Ligand-2 Single Nucleotide Polymorphism in Pulmonary Tuberculosis: A Genetic Association Study from North India

**DOI:** 10.1155/2020/1019639

**Published:** 2020-12-16

**Authors:** Sanjay K. Biswas, Mayank Mittal, Ekata Sinha, Vandana Singh, Nidhi Arela, Bharat Bajaj, Pramod K. Tiwari, Vishwa M. Katoch, Keshar K. Mohanty

**Affiliations:** ^1^Immunology Division, National JALMA Institute for Leprosy and Other Mycobacterial Diseases (Indian Council of Medical Research), Dr Miyazaki Marg, TajGanj, 282 004, Agra, India; ^2^State TB Demonstration and Training Centre, Agra, India; ^3^Department of Human Genetics, Jiwaji University, Gwalior, India; ^4^Rajasthan University of Health Sciences (RUHS), Jaipur, India; ^5^JIPMER, Puducherry, India

## Abstract

The C-C motif chemokine ligand-2 (CCL2) was evidenced to be associated with tuberculosis susceptibility in some ethnic groups. In the present study, effort was made to find out the association of *CCL2*-2518 A>G and -362 G>C variants with susceptibility to TB in a population from North India. The genotyping was carried out in 373 participants with pulmonary TB (PTB) and 248 healthy controls (HCs) for *CCL2-*2518 A>G and -362 G>C polymorphisms by PCR-RFLP and by melting curve analysis using fluorescence-labeled hybridization fluorescent resonance energy transfer (FRET) probes, respectively, followed by DNA sequencing in a few representative samples. Genotype and allele frequencies were compared by the chi-squared test and crude and Mantel-Haenszel (M-H) odds ratio (OR). OR was calculated using STATA/MP16.1 software. Further, CCL2, IL-12p70, IFN-*γ*, TNF-*α*, and TGF-*β* levels were measured in serum samples of these participants using commercially available kits. Our analysis indicated that the homozygous mutant in both -2518 GG (OR = 2.07, *p* = 0.02) and -362 CC (OR = 1.92, *p* = 0.03) genotypes was associated with susceptibility to pulmonary TB. Further, heterozygous genotypes -2518AG (OR = 0.60, *p* = 0.003) and -362GC (OR = 0.64, *p* = 0.013) provide resistance from PTB disease. Haplotype analysis revealed AC haplotype (*p* = 0.006) to be a risk factor associated with PTB susceptibility. The serum CCL2 level was significantly elevated among participants with -2518 AA genotype compared to -2518 GG genotype. CCL2 level was observed to be positively correlated with IL12p70, IFN-*γ* and TNF-*α*, thus suggesting the immunological regulatory role of CCL2 against pulmonary tuberculosis. *CCL2*-2518 GG and -362 CC genotypes were found to be associated with susceptibility to pulmonary tuberculosis and *CCL2*-2518AG and *CCL2*-362GC with resistance from PTB. AC haplotype was found to be a risk factor for PTB in the present study. It may be hypothesized from the findings that -2518G allele could be responsible for lower production of CCL2 which leads to defective Th1 response and makes a host susceptible for pulmonary tuberculosis.

## 1. Introduction

Tuberculosis (TB) is a major health concern all over the world. Globally, approximately 10 million people fell ill with TB in 2018 from the range of 5 to 500 cases per 100000 populations, out of which 57% were men, 32% were women, and 11% accounted for children less than age of 15 years; among all, 1.2 million died of TB [1]. Geographically, eight countries accounted for 2/3rd of global TB burden with India being the highest at 27% and South Africa being the lowest at 3% (the 2019 edition of the global TB report was released on 17 October 2019) (http://www.who.int/tb/data).

Susceptibility to infectious diseases after exposure to pathogen is a complex mechanism which involves the interactions among host, pathogens, and environmental factors [2]. Many studies have supported the crucial role of host genetic factors in susceptibility to PTB [3]. On exposure to *M. tuberculosis*, our first line of defense comes into play which activates our adaptive immunity which is mainly driven by CD4+ T cells and macrophages, supported by a network of inflammatory cytokines (IFN-*γ* and TNF-*α*) and chemokines. Chemokines are small molecular weight proteins involved in immunoregulatory and inflammatory functions [4], and based on their N-terminal cysteine residues, they are categorized into the following: C–, C–C, C–X–C, and C–X3–C subfamilies [5]. *CCL2*, a strong chemotactic and proinflammatory chemokine belonging to the C-C family, has been reported to provide protection against *M. tuberculosis* [6] and is reported to be stimulated by TNF-*α* along with the activation of macrophages [7–8]. The chemokine gene can be mapped in human chromosome 17q11-17q12, the two known polymorphisms -2518 A>G (rs 1024611) and -362G/C (rs 2857656) are reported in the promoter region, and the mutation in these regions affects the gene expression and has also been linked to tuberculosis susceptibility [9].

Various studies worldwide have been conducted to understand the effect of the mutation in these variants with respect to the susceptibility or resistance to pulmonary tuberculosis (PTB). The very first study in this respect was conducted by Flores-Villanueva in a Mexican population where they have reported the odds of developing pulmonary tuberculosis to be 2.3- and 5.4-fold in carrier of AG and GG genotypes, respectively, than in homozygous AA. They also reported GG to have the highest level of plasma *CCL2* and the lowest level of plasma IL-12p40 [10]. A study on population from Ghana and Russia reported -2518G and -362C to be more prevalent in control groups compared to the PTB cases, hence indicating the protective effect of the alleles against the PTB disease in a Ghanian population; on the other hand, they did not find any association in a Russian population [11]. Another study from Mexico and Peru reported that the joint effect of *CCL2*-2518GG genotype along with *MMP1*-1607GG increased the risk of developing PTB by 3.59-fold in Mexican and 3.9-fold in a Peruvian population, respectively [12]. Arji et al. [13] have reported higher prevalence of *CCL2*-2518G allele in a healthy Moroccan population suggesting a potential protective effect of the allele against the PTB disease. A meta-analysis conducted by Gong et al. [14] revealed that the G allele of the *CCL2*-2518 polymorphism is a risk factor for PTB in Asian and Americans but not Africans and the C allele of the -362G>C polymorphism is a protective factor for tuberculosis in these populations. A study conducted on a Sahariya tribe, from India, analyzed the -2518A>G and -362G>C polymorphism on PTB cases and healthy controls but they did not find any association with PTB disease [15]. Another study from a South Indian population reported a significantly decreased frequency of *CCL2*-2518GG genotype in male patients with PTB and a significantly increased frequency of the same genotype among female patients with PTB. Their results suggested that the -2518GG genotype may be associated with protection in males and susceptibility to PTB in females [16]. In a recent meta-analysis, an association between the *CCL2*-2518A>G polymorphism and human TB susceptibility was reported [17]. Earlier studies conducted on a population from Hong Kong [18] and South Africa [19] could not find any significant association with the disease. These two polymorphisms (-2518A>G and -362G>C) of *CCL2* located in the promoter region of the gene are known to play important role in immune gene regulation. The divergence in the earlier worldwide reports and in an Indian population evoked us to analyze these polymorphisms in the north Indian population from Agra, India.

So, the present study was conducted with two main objectives, i.e., to address the association of *CCL2*-2518 A>G and -362 G>C polymorphisms and haplotypes with TB in a population of the northern part of India and to analyze the correlation between the level of serum *CCL2* and cytokines in TB cases and controls with respect to their genotypes.

## 2. Materials and Methods

### 2.1. Study Subjects

The present study conducted was a part of the major project going on, in the institute which was approved by the institute's human ethical committee, constituted following the guidelines laid by Indian Council of Medical Research, New Delhi [20]. Before the start of the study, an interview schedule was formulated regarding the demographic details of the cases and controls along with the written informed consent and these were also approved by the institute's ethical committee. These written informed consents were obtained from all participants of the study, and for minor or children below 18 years of age, the written informed consent was obtained from a parent or guardian. 373 pulmonary tuberculosis cases (PTB) (mean age 32.47 ± 12.94; male : female 253 : 120) and 248 healthy controls (mean age 33.71 ± 12.82; male : female 122 : 126) were included in the study. We analyzed the *CCL2*-2518 A>G polymorphism in 373 PTB cases and 248 healthy controls and the *CCL2*-362 G>C polymorphism in 330 PTB cases and 235 healthy controls. We collected the information regarding the subject's age, sex, smoking habits, drinking habits, and BCG vaccination with the help of the interview schedule for both the cases and controls.

### 2.2. Pulmonary Tuberculosis Patients (PTB)

Cases with pulmonary TB were included in the study with in the age group of 16 to 63 years. PTB cases were recruited from the outpatient department (OPD) of the State Tuberculosis Demonstration Centre (STDC), Agra, during the period from 2007 to 2012 who were registered in the OPD on Monday, Wednesday, and Friday and met the inclusion and exclusion criteria of the PTB cases and agreed to participate in the study. The cases were mainly the residents of Agra or nearby area, within the state of Uttar Pradesh. The cases were recruited on the basis of defined clinical criteria, including the standard respiratory symptoms (fever, cough, expectoration, and malaise). The sputum smear and/or culture positivity were diagnosed on the basis of acid fast bacillus (AFB) smear positivity by Zeil-Neelsen staining and clinical symptoms following the guidelines of the Revised National TB Control Programme (RNTCP) [21]. As a routine, two sputum samples were collected over 2 days (on spot/morning sputum) and, by definition, a new smear-positive pulmonary TB case was diagnosed only when any of the sputum sample showed smear-positive result. AFB culture was performed in Lowenstein-Jensen (LJ) slant, and *M. tuberculosis* was confirmed by biochemical tests following the protocol described in Vestal [22]. We excluded all the cases showing symptoms of other form of tuberculosis, seropositive for HIV infection, or any other immunosuppressive disorders like diabetes mellitus from the study and those who have taken anti-TB drugs before.

### 2.3. Healthy Controls

Healthy controls included those subjects who escorted PTB cases to the hospital but were not blood related to the cases; randomly selected healthy subjects who were residing in the same area as those of patients, by a house-to-house survey method; between the age of 16 and 63 years; and postgraduate students who were short-term trainees in the institute and who agreed to participate in the study and also met the inclusion criteria of controls. Those with a recent history of fever, viral infection, other illness, or any other immunological disease and who have undergone treatment for tuberculosis or leprosy in the past and any family history of tuberculosis and persons found to be positive for AFB smear tests were excluded from the study. The controls were inoculated with 0.1 ml (5 tuberculin units) PPD antigen intradermally, and induration was noted after 48 to 72 hours of application in 104 healthy controls; among them, 34 (32.69%) were positive and 70 (67.30%) were negative for PPD. The detailed description of PTB cases and healthy controls is given in Table 1.

### 2.4. Collection of Blood Samples and DNA Extraction

A total of 4 ml of blood was collected from each subject where 2 ml of blood was collected in tubes containing acid citrate dextrose (ACD) from which DNA was isolated following the user's instruction using the DNA isolation kit (Midi prep from Qiagen, Germany). Another 2 ml of blood was collected in tubes without anticoagulants for separating the serum, and the separated serum was stored at -20°C with protease inhibitor for *CCL2* and other serum cytokine assays.

### 2.5. Selection of Single Nucleotide Polymorphism (SNP) and Sample Size

*CCL2*-2518 A>G and -362 G>C polymorphisms were reported to be associated with susceptibility or resistance to TB in various populations of the world [6–19]. However, a reported variation in the results and the fact that the two polymorphisms of *CCL2*-2518A>G and -362G>C located in the promoter region of the gene which plays important role in immune regulatory mechanisms induced us to analyze the polymorphism in the north Indian population from Agra, India. So, in the present study, we intended to address the association of *CCL2*-2518 A>G and -362 G>C polymorphisms with TB in a population of the northern part of India along with the level of serum *CCL2* and cytokines. Initially, a small pilot study was carried out with small sample size and after positive results were found, the sample size was calculated by statistical methods.

### 2.6. Genotyping of -2518 A>G and -362 G>C Single Nucleotide Polymorphisms

Genotyping of the *CCL2*-2518 A>G polymorphism was carried out using a polymerase chain reaction-restriction fragment length polymorphism (PCR-RFLP) method as described previously by Flores-Villanueva [10]. The region containing the -2518 A>G polymorphism in the *CCL2* promoter region was amplified using 100 ng of genomic DNA by the forward primer 5′-GCTCCGGGCCCAGTATCT-3′ and reverse primer 5′-ACAGGGAAGGTGAAGGGTATGA-3′. Restriction enzyme *PvuII* was used for the detection of *CCL2* alleles, where the allele G was represented by generation of two fragments of 182 bp and 54 bp after digestion and the allele A was identified by the presence of a 236 bp undigested fragment. These fragments were resolved on agarose gel electrophoresis.

Genotyping for -362G>C polymorphism was performed by melting curve analysis using fluorescence-labeled hybridization probes (TIB Mol Biol, Berlin, Germany) using the Light Cycler 480 system (Roche Diagnostics, Berlin, Germany) following the modified protocol of Thye et al. [11]. 5′-GAGCCTGACATGCTTTCATCTA-3′ sense primer and 5′-TTTCCATTCACTGCTGAGAC-3′ and antisense primer along with FRET probes 5′-TTCGCTTCACAGAAAGCAGAATCCTTA-3′ (3′ labeled with fluorescein) and 5′-AAATAACCCTCTTAGTTCACATCTGTGGTCAGTCT-3′ (5′ labeled with LCRed640). PCR was performed using 1.5 *μ*l DNA primers (sense and antisense) at 1.25 pmol, 2.5 mM MgCl_2_, and 250 nM of the sensor probe and anchor probe. The sensor probe was labeled with fluorescein at the 3′ end. The anchor probe was labeled with Light Cycler Red 640 at the 5′ end. Difference in temperature of melting peaks determined the different homozygous and heterozygous genotypes.

### 2.7. DNA Sequencing

The region covering both the polymorphisms, -2518 A>G and -362 G>C, of the *CCL2* gene was amplified in 10 samples from each genotype using sequence-specific primers reported previously [10, 11] using the ABI Big Dye Terminator v2 kit (Applied Biosystems, Foster City, CA, USA) in conjunction with the ABI-recommended protocol in the ABI 3700 capillary sequencer.

### 2.8. Estimation of CCL2, IL-12p70, IFN-*γ*, TNF-*γ*, and TGF-*β*

Serum CCL2 and IL-12p70, IFN-*γ*, TNF-*γ*, and TGF-*β* cytokines were assayed using respective human Duoset enzyme-linked immunosorbent assay (ELISA) Development System (R&D Systems, Minneapolis, MN, USA) in 120 tuberculosis cases (40 representative cases each from wild, heterozygous, and mutant genotypes) and 54 healthy controls (20 representative healthy controls each from heterozygous and wild genotypes and 14 from mutant genotypes with respect to the *CCL2*-2518A>G polymorphism).

### 2.9. Statistical Analysis

Allele and genotype frequencies of each polymorphism were determined by direct counting. Hardy-Weinberg equilibrium (HWE) was examined in controls and patients by *χ*^2^ test. Genotype and allele frequencies were compared between patients and controls by the chi-squared test; magnitude of association was expressed as odds ratio (OR) with 95% CI. *p* < 0.05 was considered significant for all analyses. Genotypic associations for dominant, recessive, and overdominant models were tested using STATA/MP16.1 software (StataCorp LP Lakeway Drive, College Station, TX, USA). Mantel-Haenszel (M-H) estimate was also calculated after adjusting for sex. Both crude and M-H estimates were represented.

Analysis of linkage disequilibrium (LD) and haplotype between the SNPs was carried out using online software SNP stats. Levels of serum CCL2 and cytokines were compared either using Mann-Whitney or Kruskal-Wallis tests. The Spearman rank correlation test was performed using STATA/SE 11.0 software.

## 3. Results

### 3.1. Demographic Parameter Analysis

A total of 373 PTB cases and 248 healthy controls (HCs) between the age group of 16 and 63 years were included in the present study. The mean age of PTB cases (32.47) and healthy controls (33.71) did not differ significantly; however, the male to female ratio was observed to be significantly higher in PTB cases compared to HCs (*p* = 0.007) (Table 1).

Out of the 373 PTB cases, AFB smear positivity could be analyzed for 370 cases and AFB culture positivity for 368 cases. A detailed distribution of AFB smear positivity and culture positivity is described in Table 1. The PTB cases were further analyzed on the basis of bacterial load (scanty +, 1+, 2+, and 3+) and also for any significant difference with respect to age, gender, and polymorphism, but we did not find any significant difference or association.

Healthy controls were also tested for the PTB disease, and none of them showed any sign of AFB smear positivity or culture positivity. Out of 248 HCs, 104 could be inoculated with PPD (purified protein derivative) of which 32.69% were found to be PPD positive and 67.30% were PPD negative. PPD-positive HCs were followed till the duration of the study, and none of them developed the active form of PTB.

### 3.2. Genotypic Analysis of *CCL2*-2518 A>G (rs1024611) and -362 G>C (rs2857656) Single Nucleotide Polymorphisms

*CCL2*-2518A/G polymorphism was analyzed in 373 PTB cases and 248 HCs. The frequencies of genotypes and alleles were in Hardy-Weinberg equilibrium in both cases and controls (*p* > 0.05). The male : female ratio is significantly higher in PTB cases compared to healthy controls; however, the frequencies of genotypes for both CCL2-2518A>G and -362G>C are not significantly different between males and females (*p* = 0.81 and 0.93). On comparing the genotypic frequencies of PTB cases and healthy controls, a significant difference was observed with respect to heterozygous AG genotype which was found to be significantly higher in controls (0.43, *p* < 0.003) and also the homozygous GG genotype which was found to be significantly higher in PTB patients (0.11, *p* = 0.004). A allele was found to be the dominant allele with frequency of 0.73 and 0.72 in both cases and controls, respectively. They did not differ significantly (*p* value = 0.79). On observing the significant difference in genotypic frequencies, we analyzed the association of the genotypes with disease using various models and observed that in the overdominant model, the heterozygous AG genotype was providing resistance against PTB disease [OR = 0.60 (95%CI = 0.43-0.84), *p* value = 0.003]. On the other hand, in the recessive model, the homozygous recessive genotype GG showed nearly twofold risk of developing the disease [OR = 1.97 (95%CI = 1.06-3.64), *p* value = 0.02] and M-H estimates after adjusting for sex [OR = 2.07 (CI = 1.10-3.91)] (Table 2).

*CCL2*-362G>C polymorphism was analyzed in 330 PTB cases and 235 HCs. The polymorphism was in Hardy-Weinberg equilibrium in both PTB cases and healthy controls (*p* > 0.05). Significant difference in the frequency of homozygous CC and heterozygous GC genotypes was observed for the *CCL2*-362 G>C polymorphism (rs 2857656) between PTB cases and healthy controls. The frequency of homozygous CC genotype at locus -362G>C was found significantly higher in PTB cases (0.13) than that found in healthy controls (0.07) whereas the frequency of heterozygous GC genotype was found to be higher in healthy controls (0.45) than in PTB cases (0.35). On performing association analysis in the overdominant model, we found heterozygous GC genotype to be providing protection against PTB disease [OR = 0.65 (95%CI = 0.46-0.92), *p* value = 0.01], whereas, in the recessive model, homozygous CC genotype was associated with the disease susceptibility [OR = 1.87 (CI = 1.03-3.38), *p* value = 0.03] (Table 2). The PTB cases were further categorized on the basis of bacterial load (scanty +, 1+, 2+, and 3+) and gender but we did not find any significant difference or association on categorization with any of the two polymorphism or their genotypes.

### 3.3. Haplotype Frequency and Linkage Disequilibrium Analysis of -2518 A>G (rs1024611) and -362 G>C (rs2857656) Loci of *CCL2* among PTB Cases and Healthy Controls

1090 chromosomes were studied for haplotype analysis for these two loci of chromosome 17q11-17q12. All the four haplotypes were present in the studied population. The frequency of AG (0.70) haplotype was higher compared to that of GC (0.25), AC (0.04), and GG (0.01) haplotype in PTB cases. The frequency of AG (0.68) haplotype was higher among the healthy controls compared to GC (0.28), AC (0.004), and GG (0.002) haplotype. The frequency of AC haplotype was found to be significantly high (0.046) in PTB cases compared to HCs (0.004) [OR = 7.23 (CI = 1.74-30.09), *p* value = 0.006] (Table 2). A strong LD was observed between both the sites (*D*′ = 0.961, *p* value = 0.00) indicating that the studied loci were in linkage disequilibrium with each other in the present population.

### 3.4. Serum Analysis of CCL2, IL-12p70, IFN-*γ*, TNF-*α*, and TGF-*β*

**Figure 1:**
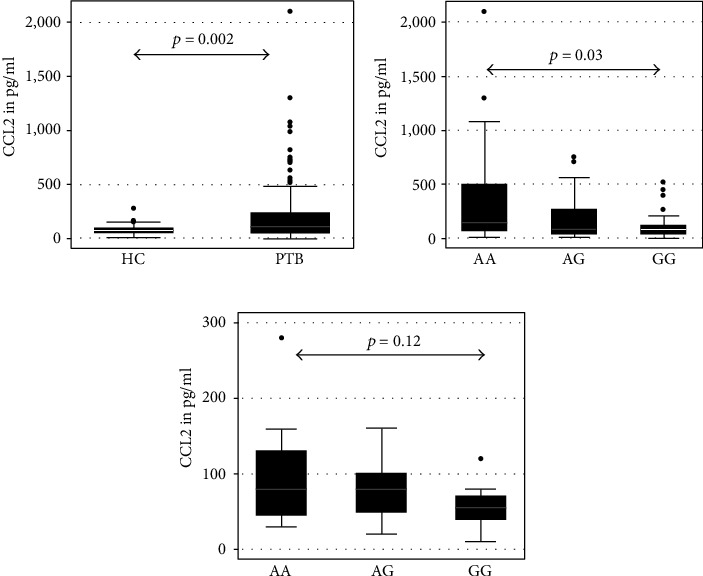
Comparative analysis of serum CCL2 level in PTB patients and healthy controls. Serum CCL2 was measured by sandwich ELISA using Duoset from R&D Systems, USA, in pulmonary TB patients and controls. The level was expressed as pg/ml on the *Y*-axis and represented as box and whisker plot. Each dot above the vertical box plot represents the outside value of one subject. Each box is with whiskers on both sides with upper and lower adjacent values, respectively. The box shows the 75^th^, median, and 25^th^ percentile values from the upper hinge to lower hinge, respectively. Subjects are represented on the *X*-axis. HC: healthy controls; PTB: pulmonary TB patients; AA: subjects with CCL2-2518AA genotypes; AG: subjects with CCL2-2518AG genotype; GG: subjects with CCL2-2518GG genotypes. (a) For comparison of CCL2 level between HC and PTB patients. (b) For comparison of the CCL2 level among PTB patients having various CCL2 genotypes. (c) The comparison of the CCL2 level among HC having various genotypes. Pairwise comparison was made by the Wilcoxon rank-sum test, and comparison of groups was done by the Kruskal-Wallis equality of population rank test. *p* values are shown above the box plots.

CCL2/MCP-1, IL12p70, IFN-*γ*, TNF-*α*, and TGF-*β* were measured in serum samples of 120 pulmonary TB patients and 54 healthy controls. The CCL2 level was observed to be significantly elevated in PTB patients compared to healthy controls (*p* < 0.005) (shown in Figure 1) and varied among CCL2 variants in PTB patients (Spearman corr = −0.225, *p* = 0.01). The significantly higher mean level of serum CCL2 was observed in cases with -2518 homozygous AA genotype (352.8 pg/ml) compared to the level seen in cases with -2518 heterozygous AG genotype (183.3 pg/ml) and -2518 homozygous GG genotype (119.3 pg/ml) (*p* = 0.03).

**Figure 2:**
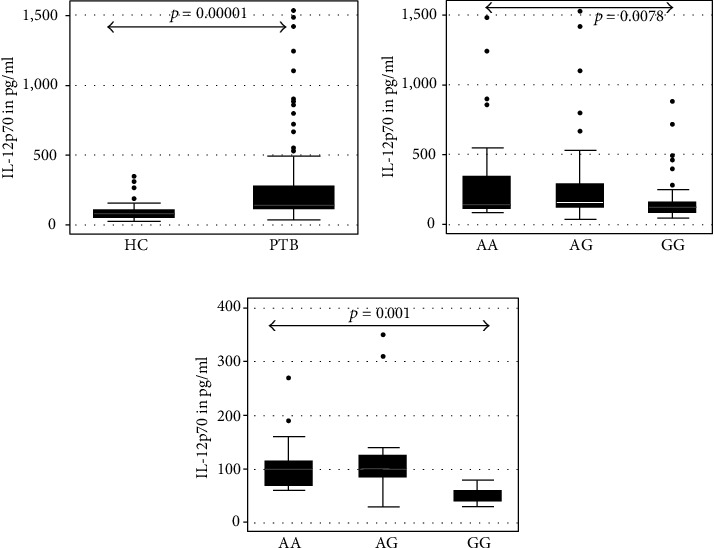
Comparative analysis of serum IL-12p70 level in PTB patients and healthy controls. Serum IL-12p70 was measured by sandwich ELISA using Duoset from R&D Systems, USA, in pulmonary TB patients and controls. The level was expressed as pg/ml on the *Y*-axis and represented as box and whisker plot. Each dot above the vertical box plot represents the outside value of one subject. Each box is with whiskers on both sides with upper and lower adjacent values, respectively. The box shows the 75^th^, median, and 25^th^percentile values from the upper hinge to lower hinge, respectively. Subjects are represented on the *X*-axis. HC: healthy controls; PTB: pulmonary TB patients; AA: subjects with CCL2-2518AA genotypes, AG: subjects with CCL2-2518AG genotype; GG: subjects with CCL2-2518GG genotypes. (a) For comparison of IL-12p70 level between HC and PTB patients. (b) For comparison of IL-12p70 level among PTB patients having various CCL2 genotypes. (c) The comparison of IL-12p70 level among HC having various genotypes. Pairwise comparison was made by the Wilcoxon rank-sum test, and comparison of groups was done by the Kruskal-Wallis equality of populations rank test. *p* values are shown above the box plots.

The serum IL-12p70 level was found to be significantly higher in PTB patients compared to healthy controls (*p* = 0.0000) (shown in Figure 2) and differed significantly among subjects having various genotypes of *CCL2*-2518 variants (*p* < 0.05); it was observed to be significantly higher in PTB patients with homozygous AA and heterozygous AG genotypes compared to homozygous GG genotypes.

The serum CCL2 level is significantly positively correlated with the serum level of IL-12p70 in healthy controls as well as in PTB cases. On analysis with reference to specific genotypes of the *CCL2*-2518 A>G variant, the serum CCL2 level was observed to be significantly positively correlated in healthy controls, having homozygous AA genotypes (Spearman *r* = 0.79, *p* = 0.000) and heterozygous AG genotypes (*R* = 0.68, *p* = 0.0009), with the serum IL-12p70, whereas in PTB cases, the serum CCL2 level was observed to be significantly positively correlated with the serum IL-12p70 in homozygous GG genotype (Spearman rho = 0.51, *p* = 0.0008) only.

Th1 cytokines IFN-*γ* and TNF-*α* were also analyzed in relation to the *CCL2*-2518 A>G variant and correlated with serum CCL2. A positive significant correlation was found.

Regression analysis of CCL2 with all these cytokines revealed that the level of serum CCL2 was significantly correlated with the level of serum IL-12p70 (regression coefficient 0.37, *p* = 0.048) and IFN-*γ* (regression coefficient = 1.69, *p* = 0.00) in healthy controls.

## 4. Discussion

The present study explored the genetic frequencies of *CCL2*-2518 A>G and -362 G>C polymorphisms in a north Indian population from Agra, India. Pulmonary TB cases and healthy controls were recruited keeping in mind their similar environmental exposure and socioeconomic background. Although a number of reports have been published suggesting the role of *CCL2* gene polymorphism in different populations, the findings are often contradictory based on the ethnicity [23], population [24], and type of tuberculosis [25, 26]. Other studies reported from India are based on a south Indian population [27] and in tribal population [15]. Our study is based on a population from the northern part of India, and no report on the *CCL2* gene polymorphism is available for this region in relation to tuberculosis. The participants for this study were included between 2007 and 2012, as tuberculosis is prevalent in this part which was the persuasive situation for this study to be carried out. In comparison to other studies, we have attempted to partially address the functional relevance of this polymorphism with reference to its possible regulatory role in cytokine levels. In our observations -2518A allele and -2518AA genotype are noted to be predominant allele and genotype, respectively, in the present population. Flores-Villanueva et al., in a Mexican population, reported -2518 G allele and GG genotype to be the major allele and genotype, respectively, in their study [10]. They have reported in their study a 5.4- and 6.9-fold increased risk of developing TB in the carrier of GG genotype in a Mexican and Korean population, respectively; we also found the same, i.e., association of *CCL2*-2518 GG genotype with susceptibility to TB (*p* = 0.02) in the recessive model (Table 2). Our result of AG genotype was totally opposite as reported by them where they found 2.3- and 2.8-fold increased risk of developing the disease in a Mexican and Korean population, respectively, and we found in the present study that -2518 AG was providing resistance to the disease (*p* value = 0.003) (Table 2). Other population studies too have reported association with G allele of -2518 polymorphism; Gong et al. [14] have reported in their meta-analysis that G allele of -2518 polymorphism is a risk factor for TB in Asian and American population but not in African population. Thye et al. [11] and Arji et al. [13] have reported the protective role of G allele of -2518 polymorphism in a Ghanian and Moroccan population, respectively. A study conducted on a Sahariya tribe (the tribe is reported to have high TB prevalence) from India did not report any association with *CCL2*-2518A/G polymorphism [15]. Another study from the mainland of India, on a South Indian population, reported association of *CCL2*-2518 GG genotype with protection against PTB in males but in contrast, at the same time, they reported it to be susceptible for developing the disease in females [16]. In the present study, we found the GG genotype of -2518 to be having approximately 2-fold increased risk of developing PTB.

The other variant of *CCL2* reported worldwide is -362 G>C; in the present study, we found the *CCL2*-362 GC genotype to be significantly high among healthy controls (*p* = 0.01) compared to PTB cases suggesting a protective role of the genotype against PTB; on the other hand, the homozygous -362 CC genotype is found to be a risk genotype (Table 2); this is in contrast to the report by Thye et al. [11] where they reported that both CC and CG genotypes were overrepresented in healthy controls in a Ghanaian population and the -362C allele was associated with protection against TB. Mishra et al. [15] could not find a significant difference in frequencies of allele or genotypes of -362 G>C polymorphism among the PTB cases and HCs of a primitive tribal group “Saharia,” although the GC genotype was more frequently present in controls. Velez Edwards et al. [28] also could not find any evidence of association of the -362 G>C polymorphism with PTB in Guinea Bissau, the Gambian, and African-American populations. Thye et al. reported a significant heterogeneity in association of the -362 G>C polymorphism with PTB between studies in meta-analysis of five case control studies from five ethnicities [11]. The ethnic variation could be the reason for difference in observations.

Our study subjects were drawn from a population consisting of multireligion communities residing near Agra in Uttar Pradesh and nearby states. Here, we found that the GG genotype of *CCL2* 2518 A>G is overrepresented in PTB patients. While AG genotype is higher in healthy controls (both tuberculin-positive and tuberculin-negative controls) compared to PTB patients. There was no differences in genotype frequencies between tuberculin-positive and tuberculin-negative individuals, and the frequency of PPD (+) and PPD (-) individuals was the same as the national frequency. It is noteworthy to find the heterozygous genotype providing protection from the disease. Both the polymorphisms -2518 A>G and -362 G>C provided protection against the disease in a heterozygous condition. The heterozygous protection is represented by the overdominance model and the model states that polymorphism is maintained because heterozygous individuals are able to recognize a wider variety of parasites [29], and India being the TB endemic region, we can speculate that this heterozygous effect played some role in local adaptation against TB; it has been previously described by Sinha et al. [30]. To substantiate our hypothesis, we further analyzed the functional aspect of CCL2 in serum. We detected a significantly higher level of serum CCL2 in PTB patients compared to healthy controls which is in accordance with the findings of earlier studies [4, 5]. In contrast to their observations, our findings indicate a strong association of the serum CCL2 level with various genotypes of -2518 A>G polymorphism in PTB cases. PTB cases with the -2518AA genotype showed a significantly higher level of serum CCL2 compared to the cases with -2518 AG and -2518 GG genotypes. Flores-Villanueva et al. [10] and Rovin et al. [31] had noted a higher level of CCL2 in -2518 GG patients and lower serum IL-12. We observed a significantly higher level of IL-12p70 and IFN-*γ* in serum in PTB cases compared to healthy controls. The level of IL-12p70 was positively correlated to the CCL2 level in both the groups of study subjects. The level of CCL2 was significantly correlated with IL-12, IFN-*γ*, and TNF-*α* in PTB cases with the -2518 GG genotype. So, the lower level of CCL2 could be responsible for the low level of IL-12 and IFN gamma, which were evident to be protective cytokines giving resistance to TB infection. This could be the reason for -2518 GG genotype susceptibility for PTB in the present population. -2518 GG genotype cases produced less amount of CCL2 and also less IL-12p70 resulting in increased susceptibility to the disease. Hence, subjects with -2518 GG genotypes are more likely to be prone to *M. tuberculosis* infection in the present population, and the lower level of IL-12p70 could possibly influence the lower immunity of these people. Additionally, healthy people with AG genotypes showing an intermediate level of IL-12p70 probably would be regulating in some way the CCL2 level giving the protective effect against *M. tuberculosis* infection in the present population. Furthermore, for the first time, the level of other important cytokines in serum with reference to various *CCL2*-2518 A>G genotypes was analyzed in the present study. PTB subjects with the -2518GG genotype were having a lower level of IFN-*γ*, presumably due to the lower level of CCL2 and IL12p70. The TGF-*β* level was significantly higher in healthy controls compared to PTB cases. The higher level of TGF-*β* in healthy subjects with the -2518AG genotype is suggestive of its regulatory role in providing protection against TB infection.

We explored the correlation of the serum CCL2 level with IL-12p70, IFN-*γ*, TNF-*α*, and TGF-*β* in subjects with variants of -2518A>G. Regression analysis suggested that the serum CCL2 concentration regulates the concentration of IL-12p70 and more strongly IFN-*γ* concentration. Stratification on the basis of genotypes indicated that healthy subjects with the -2518AA or -2518AG genotype with a high level of CCL2 probably positively regulate the production of key cytokines IL-12p70 and IFN-*γ* which could be the reason for providing protective immunity. On the other hand, PTB cases with the *CCL2*-2518 GG genotype were having lower concentration of CCL2 thus lowering the production of IFN-*γ* and becoming susceptible to infection due to poor Th1 response. An earlier report by Velez Edwards et al. [28] observed interaction between one of the CCL2 and IL12B polymorphisms in Africans, and the effect was opposite.

It was general assumption that higher CCL2 promotes Th2 response and suppresses Th1 response. The present study for the first time showed that CCL2 has a positive correlation with IL-12 and Th1 cytokines such as IFN-*γ* and TNF-*α* and hence essential for proper Th1 response against tuberculosis. -2518G allele is responsible for lower production of CCL2 which leads to lower Th1 cytokines, hence leading to defective Th1 response which makes a host susceptible for tuberculosis. Divergent observations about the genetic association with diseases across the ethnically different population are well reported. Similar nucleotide polymorphism can act differently in different environmental setups to affect the susceptibility. These include the duration of exposure to infectious agents, nutritional status of the individuals, and the other epigenetic factors. The varied observations also could be due to the genetic differences among the population and relatively small size of the database.

The C-C motif chemokine ligand 2 (CCL2) is a member of the small inducible gene (SIG) family. CC-chemokines are characterized by two adjacent cysteine residues close to the amino terminus of the molecule. They are involved in the recruitment of lymphocytes and monocytes and control migration of these cells to sites of cell injury and cellular immune reactions [32]. CCL2 is produced by different cell types in response to microbial stimuli [33]. The polymorphisms studied in the present population could have effect on each other so it becomes important to study the haplotypic analysis of the polymorphism. In the present study, AG haplotype was found to be the most predominant haplotype in both PTB cases and healthy controls. Interestingly, on haplotype analysis, AC haplotype was found to be the susceptible haplotype for tuberculosis (Table 3). The two polymorphism (-2518 A>G and -362 G>C) were in linkage disequilibrium having strong D′ between them. Thye et al. [11] have shown through interaction analysis that the *CCL2*-362 G>C variant exclusively explains the observed association with resistance to TB whereas the *CCL2* -2518 A>G variant was not independent of -362 G>C. These observations suggest that this haplotype blocks consisting of these two polymorphisms which shows that a strong LD between them has been jointly inherited in the present population to exert certain effect in the development of tuberculosis in the prevailing environmental factors. Intemann et al. [34] have reported that haplotype comprising -2581G/-362C/int1del 554-567 has stronger protection than the -362 G>C variant alone. These haplotype variants result in decreased CCL2 expression and decreased risk of TB. Ganachari et al. [12] have reported that the haplotype consisting of *CCL2*-2518 GG along with MMP-1607 GG increases the risk of developing TB -3.5-fold in Mexican and 3.9-fold in Peruvian populations. As AC haplotype of *CCL2*-2518 A>G and -362 G> C was observed to be a susceptible haplotype for *M. tuberculosis* infection in this region, polymorphisms in these regulatory regions may be functioning in a complex manner influencing the immune function and disease susceptibility. To understand this complex interaction of the polymorphism in the present population, an in-depth immunological analysis is needed further.

The study has its own limitation such as having a comparatively smaller data set. The functional relevance of these polymorphisms with respect to immune responses to tuberculosis could be addressed in a classified manner.

## 5. Conclusion

This study reported a significant association of the *CCL2*-2518 GG and -362 CC genotype with tuberculosis. Heterozygous *CCL2-2518AG* and *-362GC* were noted to be associated with resistance against PTB. The biallelic AC haplotype (*CCL2-*2518 A>G and -362 G>C) was noted to be a susceptible haplotype for pulmonary tuberculosis. The serum cytokine analysis suggested a complex regulatory mechanism among CCL2/MCP-1, IL-12p70, and IFN-*γ* concentration. CCL2 showed a positive correlation with IL-12, IFN-*γ*, and TNF-*α*, and a normal CCL2 level is essential for normal Th1 response. -2518G allele produces less CCL2 compared to A allele which leads to improper Th1 response and makes a host susceptible for tuberculosis.

In the present study, we have tried to unwind the function of the gene in the promoter region of the *CCL2* gene but an in-depth study is warranted to further understand the complex interaction of the polymorphisms in the regulatory region of *CCL2*.

## Figures and Tables

**Table 1:** Characteristics of pulmonary TB cases and healthy controls included in the study.

	Pulmonary TB cases *N* = 373	Healthy controls *N* = 248	*p* value
Age mean ± SD	32.47 ± 12.94	33.71 ± 12.82	0.24^∗^
Gender (male : female)	253 : 120	122 : 126	0.00003^∗∗^
AFB smear positivity		ND	
1+	107		
2+	85		
3+	134		
Scanty	19		
X-ray positive	25		
Not known	3		
AFB culture positivity		ND	
1+	99		
2+	99		
3+	17		
Scanty	153		
Not known	5		
PPD status	ND		
Positive		34 (32.69%)	
Negative		70 (67.30%)	

^∗^ *p* value for *t*-test, ^∗∗^*p* value for *χ*^2^ test. PPD test was carried out in 104 healthy control subjects. Percentage of positivity was calculated among these subjects.

**Table 2:** Genotype analysis of CCL2-2518 A>G and-362 G>C polymorphisms in PTB cases and healthy control.

Genotype/allele	PTB cases	Healthy controls	Chi (DF)	OR (95% CI)	*p* value
-2518 A>G	*N* = 373 No (frequency)	*N* = 248 No (frequency)			
AA	214 (0.57)	126 (0.51)			
AG	117 (0.31)	107 (0.43)			
GG	42 (0.11)	15 (0.06)	11.31 (2)		0.004
A allele	545 (0.73)	359 (0.72)			
G allele	201 (0.27)	137 (0.28)	0.07 (1)	0.0.97 (0.74-1.24)	0.79
		MH-OR (Adj. for sex)	0.02 (1)	0.98 (0.75-1.27)	0.88
Dominant model					
AA	214 (0.57)	126 (0.51)
AG+GG	159 (0.43)	122 (0.49)	2.59 (1)	0.76 (0.55-1.06)	0.10
		MH-OR (Adj. for sex)	2.32 (1)	0.77 (0.55-1.07)	0.12
Overdominant model					
AA+GG	256 (0.69)	141 (0.57)			
AG	117 (0.31)	107 (0.43)	8.95 (1)	0.60 (0.43-0.84)	0.003
		MH-OR (Adj. for sex)	8.81 (1)	0.60 (0.42-0.84	0.003
Recessive model					
AA+AG	331 (0.89)	233 (0.94)			
GG	42 (0.11)	15 (0.06)	4.85 (1)	1.97 (1.06-3.64)	0.028
		MH-OR (Adj. for sex)	5.35 (1)	2.07 (1.10-3.91)	0.02
-362 G>C	*N* = 330	*N* = 235			
GG	174 (0.53)	113 (0.48)			
GC	114 (0.35)	105 (0.45)			
CC	42 (0.13)	17 (0.07)	8.18 (2)		0.017
G allele	462 (0.70)	331 (0.70)			
C allele	198 (0.30)	139 (0.30)	0.02 (1)	1.02 (0.78-1.3)	0.87
		MH-OR (Adj. for sex)	0.05 (1)	1.03 (0.79-1.33)	0.82
Dominant model					
GG	174 (0.53)	113 (0.48)			
GC+CC	156 (0.47)	122 (0.52)	1.18 (1)	0.83 (0.59-1.16)	0.27
		MH-OR (Adj. for sex)	1.13 (1)	0.83 (0.59-1.16)	0.28
Overdominant model					
GG+CC	216 (0.65)	130 (0.55)			
GC	114 (0.34)	105 (0.45)	5.93 (1)	0.65 (0.46-0.92)	0.015
		MH-OR (Adj. for sex)	6.15 (1)	0.64 (0.45-0.91)	0.013
Recessive model					
GG+GC	288 (0.87)	218 (0.93)			
CC	42 (0.13)	17 (0.07)	4.42 (1)	1.87 (1.03-3.38)	0.035
		MH-OR (Adj. for sex)	4.89 (1)	1.92 (1.06-3.47)	0.027

**Table 3:** Haplotype analysis for -2518 A>G and -362 G>C polymorphism in PTB cases and controls.

	-2518 A>G SNP	-362 G>C SNP	PTB cases *N* = 329	Controls *N* = 216	OR (95% CI)	*p* value
1	A	G	0.706	0.688	1	—
2	G	C	0.254	0.287	0.94 (0.72-1.23)	0.66
3	A	C	0.046	0.004	7.23 (1.74-30.09)	0.006
4	G	G	0.011	0.002	4.12 (0.51-33.25)	0.18

SNP: single nucleotide polymorphism; *N*: total number of subjects; *p*: *p* value; OR: odds ratio; CI: confidence interval.

## Data Availability

The demographic, genotypic, haplotypic, and serological data used to support the findings of this study are included within the article itself in the form of tables and figures.

## References

[1] Global TB report 20^th^ Edition, Executive summary. http://www.who.int/tb/publications/global_report/gtbr2015_executive_summry.pdf.

[2] Möller M., Hoal E. G. (2010). Current findings, challenges and novel approaches in human genetic susceptibility to tuberculosis. *Tuberculosis (Edinburgh, Scotland)*.

[3] Möller M., Wit E. D., Hoal E. G. (2010). Past, present and future directions in human genetic susceptibility to tuberculosis. *FEMS Immunology & Medical Microbiology*.

[4] Rollins B. J. (1997). Chemokines. *Blood*.

[5] Zlotnik A., Yoshie O. (2000). Chemokines: a new classification system and their role in immunity. *Immunity*.

[6] Kipnis A., Basaraba R. J., Orme I. M., Cooper A. M. (2003). Role of chemokine ligand 2 in the protective response to early murine pulmonary tuberculosis. *Immunology*.

[7] Collins H. L., Kaufmann S. H. (2001). The many faces of host responses to tuberculosis. *Imunology*.

[8] Algood H. M., Chan J., Flynn J. A. L. (2003). Chemokines and tuberculosis. *Cytokine & Growth Factor Reviews*.

[9] Jamieson S. E., Miller E. N., Black G. F. (2004). Evidence for a cluster of genes on chromosome 17q11-q21 controlling susceptibility to tuberculosis and leprosy in Brazilians. *Genes and Immunity*.

[10] Flores-Villanueva P. O., Ruiz-Morales J., Song C. H. (2005). A functional promoter polymorphism in monocyte chemoattractant protein is associated with increased susceptibility to pulmonary tuberculosis. *JEM 2005*.

[11] Thye T., Nejentsev S., Intemann C. D. (2009). MCP-1 promoter variant −362C associated with protection from pulmonary tuberculosis in Ghana, West Africa. *Human Molecular Genetics*.

[12] Ganachari M., Ruiz-Morales J. A., Gomez de la Torre Pretell J. C., Dinh J., Granados J., Flores-Villanueva P. O. (2010). Joint effect of MCP-1 genotype GG and MMP-1 genotype 2G/2G increases the likelihood of developing pulmonary tuberculosis in BCG-vaccinated individuals. *PLoS one*.

[13] Arji N., Busson M., Iraqi G. (2012). The MCP-1 (CCL2) -2518 GG genotype is associated with protection against pulmonary tuberculosis in Moroccan patients. *Journal of Infection in Developing Countries*.

[14] Gong T., Yang M., Qi L., Shen M., du Y. (2013). Association of MCP-1 -2518A/G and -362G/C variants and tuberculosis susceptibility: a meta-analysis. *Infection, Genetics and Evolution*.

[15] Mishra G., Poojary S. S., Raj P., Tiwari P. K. (2012). Genetic polymorphisms of _CCL2_ , _CCL5_ , _CCR2_ and _CCR5_ genes in Sahariya tribe of North Central India: an association study with pulmonary tuberculosis. *Infection, Genetics and Evolution*.

[16] Singh B., Chitra J., Selvaraj P. (2014). CCL2, CCL3 and CCL4 gene polymorphisms in pulmonary tuberculosis patients of South India. *International Journal of Immunogenetics*.

[17] Tian G., Li X., Li H., Wang X., Cheng B. (2015). Systematic meta-analysis of the association between monocyte chemoattractant protein-1-2518A/G polymorphism and risk of tuberculosis. *Genetics and Molecular Research*.

[18] Chu S. F., Tam C. M., Wong H. S., Kam K. M., Lau Y. L., Chiang A. K. S. (2007). Association between _RANTES_ functional polymorphisms and tuberculosis in Hong Kong Chinese. *Genes and Immunity*.

[19] Möller M., Nebel A., Valentonyte R., van Helden P. D., Schreiber S., Hoal E. G. (2009). Investigation of chromosome 17 candidate genes in susceptibility to TB in a South African population. *Tuberculosis*.

[20] Ethical guidelines for biomedical research on human participants Indian Council of Medical Research New Delhi. http://icmr.nic.in/ethical_guidelines.pdf.

[21] RNTCP Guidelines 2007-TBC India. Diagnosis of smear positive pulmonary TB. http://www.tbcindia.org.

[22] Vestal A. L. (1977). Identification test technique. *Procedure for Isolation and Identification of Mycobacteria. U.S. Department of Health, Education and Welfare Publication No. CDC-77-8230*.

[23] Vásquez-Loarte T., Trubnykova M., Guio H. (2015). Genetic association meta-analysis: a new classification to assess ethnicity using the association of MCP-1-2518 polymorphism and tuberculosis susceptibility as a model. *BMC Genetics*.

[24] Feng W.-X., Flores-Villanueva P. O., Mokrousov I. (2012). CCL2−2518 (A/G) polymorphisms and tuberculosis susceptibility: a meta-analysis. *The International Journal of Tuberculosis and Lung Disease*.

[25] Feng W.-X., Mokrousov I., Wang B.-B. (2011). Tag SNP polymorphism of CCL2 and its role in clinical tuberculosis in Han Chinese pediatric population. *PLoS One*.

[26] Nonghanphithak D., Reechaipichitkul W., Namwat W., Lulitanond V., Naranbhai V., Faksri K. (2016). Genetic polymorphisms of CCL2 associated with susceptibility to latent tuberculous infection in Thailand. *The International Journal of Tuberculosis and Lung Disease*.

[27] Alagarasu K., Selvaraj P., Swaminathan S., Raghavan S., Narendran G., Narayanan P. R. (2009). CCR2, MCP-1, SDF-1a &amp; DC-SIGN gene polymorphisms in HIV-1 infected patients with &amp; without tuberculosis. *The Indian Journal of Medical Research*.

[28] Velez Edwards D. R., Tacconelli A., Wejse et l C. (2012). MCP1 SNPs and pulmonary tuberculosis in cohorts from West Africa, the USA and Argentina: lack of association or epistasis with IL12B polymorphisms. *PLoS One*.

[29] Doherty P., Zinkernagel R. (1975). Enhanced immunological surveillance in mice heterozygous at the H-2 gene complex. *Nature*.

[30] Sinha E., Biswas S. K., Mittal M. (2014). Toll-like receptor 1 743 A>G, 1805 T>G & Toll-like receptor 6 745 C>T gene polymorphism and tuberculosis: a case control study of north Indian population from Agra (India). *Human Immunology*.

[31] Rovin B. H., Lu L., Saxena R. (1999). A novel polymorphism in the MCP-1 gene regulatory region that influences MCP-1 expression. *Biochemical and Biophysical Research Communications*.

[32] Carr M. W., Roth S. J., Luther E., Rose S. S., Springer T. A. (1994). Monocyte chemoattractant protein 1 acts as a T-lymphocyte chemoattractant. *Proceedings of the National Academy of Sciences*.

[33] Serbina N. V., Jia T., Hohl T. M., Pamer E. G. (2008). Monocyte-mediated defense against microbial pathogens. *Annual Review of Immunology*.

[34] Intemann C. D., Thye T., Förster B. (2011). MCP1 haplotypes associated with protection from pulmonary tuberculosis. *BMC Genetics*.

